# Dihydronicotinamide riboside: synthesis from nicotinamide riboside chloride, purification and stability studies†

**DOI:** 10.1039/d1ra02062e

**Published:** 2021-06-14

**Authors:** Amin Zarei, Leila Khazdooz, Mojtaba Enayati, Sara Madarshahian, Timothy J. Wooster, Gerhard Ufheil, Alireza Abbaspourrad

**Affiliations:** Department of Food Science, College of Agriculture and Life Sciences, Cornell University Ithaca 14853 NY USA alireza@cornell.edu; Institute of Material Science, Nestle Research CH-1000 Lausanne 26 Switzerland; Nestle Product Technology Center Bridgewater, Nestle Nutrition R&D Inc. Bridgewater NJ 08807 USA

## Abstract

In the present work, we describe an efficient method for scalable synthesis and purification of 1,4-dihydronicotinamide riboside (NRH) from commercially available nicotinamide riboside chloride (NRCl) and in the presence of sodium dithionate as a reducing agent. NRH is industrially relevant as the most effective, synthetic NAD^+^ precursor. We demonstrated that solid phase synthesis cannot be used for the reduction of NRCl to NRH in high yield, whereas a reduction reaction in water at room temperature under anaerobic conditions is shown to be very effective, reaching a 55% isolation yield. For the first time, by using common column chromatography, we were able to highly purify this sensitive bio-compound with good yield. A series of identifications and analyses including HPLC, NMR, LC-MS, FTIR, and UV-vis spectroscopy were performed on the purified sample, confirming the structure of NRH as well as its purity to be 96%. Thermal analysis of NRH showed higher thermal stability compared to NRCl, and with two major weight losses, one at 218 °C and another at 805 °C. We also investigated the long term stability effects of temperature, pH, light, and oxygen (as air) on the NRH in aqueous solutions. Our results show that NRH can be oxidized in the presence of oxygen, and it hydrolyzed quickly in acidic conditions. It was also found that the degradation rate is lower under a N_2_ atmosphere, at lower temperatures, and under basic pH conditions.

## Abstract 2

A convenient and scalable method for synthesis of dihydronicotinamide riboside (NRH) from the commercially available nicotinamide riboside chloride (NRCl) is elaborated as well as a fast purification method that led to high purity NRH.

## Graphical abstract

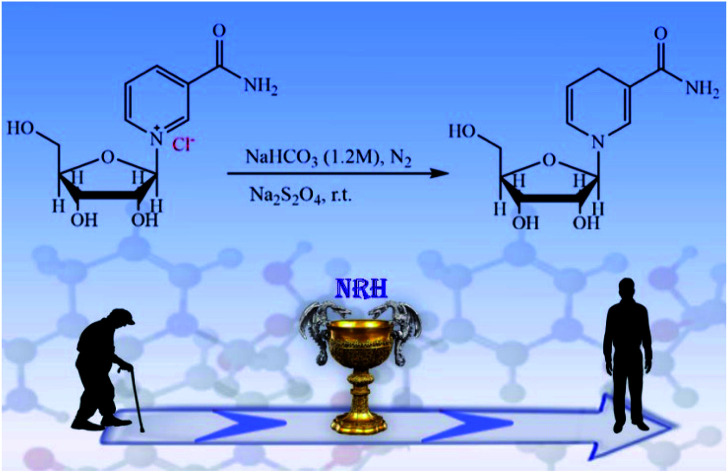

## Introduction

**Scheme 1:**
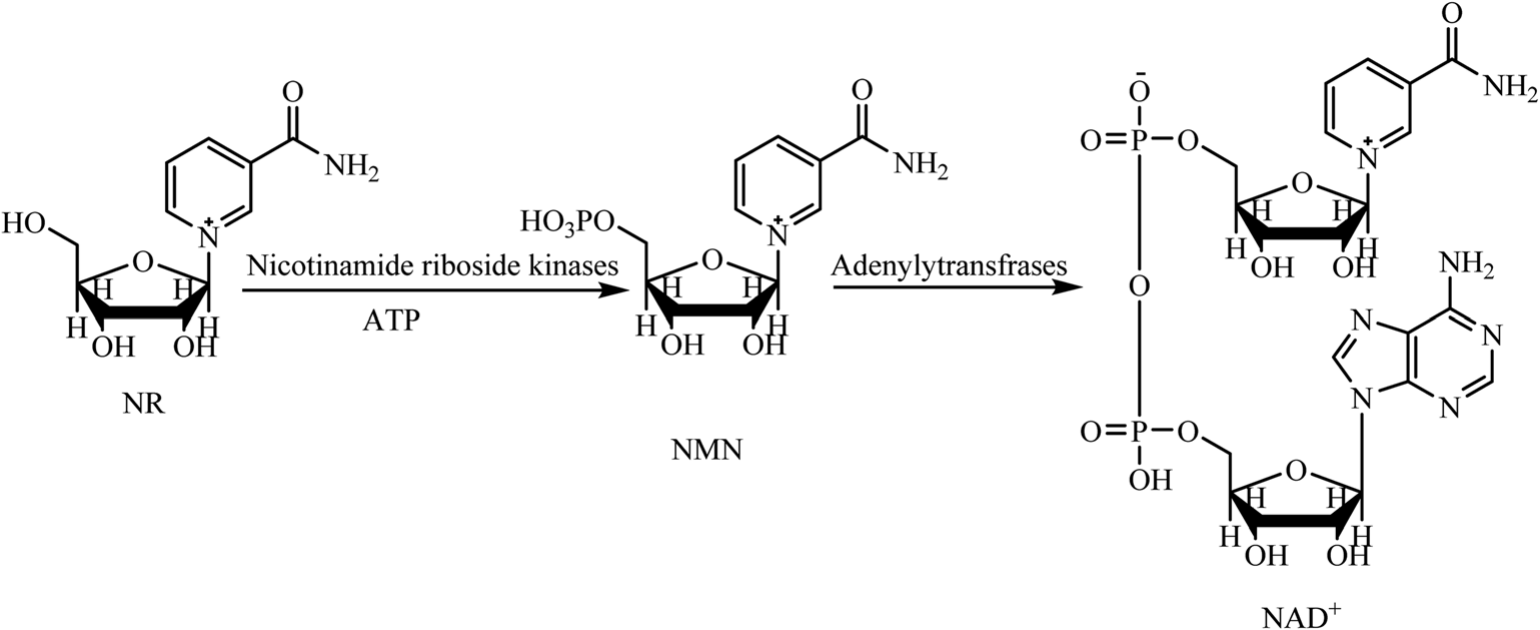
The transformation of NR into the NAD^+^ in mammalian cell.^13^

Nicotinamide adenine dinucleotide (NAD^+^), and its reduced form 1,4-dihydronicotinamide adenine dinucleotide (NADH), are key molecules in energy metabolism and mitochondrial function by electron transfer.^1,2^ Moreover, NAD^+^ is a crucial cofactor in a number of non-redox reactions, by causing adenosine diphosphate ribose (ADP-ribose) to enzymatically catalyze the functions of the two essential protein families, sirtuins (SIRTs) and poly(ADP-ribose) polymerases (PARPs).^3–5^ The sirtuins play several key roles maintaining nuclear, mitochondrial, cytoplasmic or metabolic homeostasis. The most important roles of PARPs are repairing DNA and maintaining chromatin structure and function. Aging, and some disruptive factors such as an acute injury or chronic metabolic or inflammatory conditions, can cause levels of NAD^+^ to severely decline.^6^ The drop of NAD^+^ leads to the decrease of energy production which, subsequently, impairs the cellular function, cellular homeostasis, and immune cell function.^7–10^ This phenomenon becomes even more important when the cells are damaged by ordinary environmental factors, because repairing damage requires a large amount of NAD^+^. If the injury is severe, the damaged cells will not have enough stored energy to provide the NAD^+^ needed for homeostasis maintenance, and so the damage becomes irreversible.^11^ The brain, heart, liver, kidneys and skeletal muscles are the organs with higher numbers of mitochondria, therefore, these vital organs are more susceptible to NAD^+^ depletion. Therefore, an energy-rich NAD^+^ precursor is needed to keep the cell with damaged tissue at normal levels of energy.^11,12^ Nicotinamide riboside (NR), nicotinic acid (niacin), and nicotinamide are commercially available, natural compounds used as nutritional supplements to increase the concentrations of NAD^+^. NR is a more efficient NAD^+^ precursor in comparison to niacin and nicotinamide because it is metabolized to NAD^+^ in mammalian cells in fewer steps (Scheme 1).^13^ Studies show that taking NR as a supplement is effective for stimulating NAD^+^ metabolism and can boost the level of NAD^+^ by 60 percent.^14^

Although NR can increase the NAD^+^ level of cell and improve cell health, NR must be taken in large quantities to be effective. Recently, Sauve *et al.* have synthesized 1,4-dihydronicotinamide riboside (NRH) and demonstrated that this compound is a potent NAD^+^ concentration enhancer in both *in vitro* and *in vivo* conditions.^15,16^ They found that, after administration of NRH to mammalian cells, it increased the NAD^+^ concentration by 2.5–10-fold over control values in just one hour. Their findings demonstrate that the use of NRH is more effective than either NR or NMN. Moreover, NRH considerably enhances the NAD^+^/NADH ratio in the cultured cells without induction of apoptotic markers or a substantial increases in lactate levels in cells.^15^ More recently, Canto *et al.* have found that, contrary to the NR pathway, NRH uses different steps and enzymes to synthesize NAD^+^.^17^ That explains why NRH is a more effective and a faster NAD^+^ precursor compared to the NR in mammalian cells. The same researchers have also demonstrated, in experiments with mice, that NRH is orally bioavailable as an NAD^+^ precursor and prevents cisplatin-induced, acute kidney injury.^17^ In addition to increasing NAD^+^ levels, NRH can also deplete some genotoxins such as hydrogen peroxide and methylmethane sulfonate. As a result, the mouse cells treated with NRH are resistant to cell death.^15^

**Scheme 2:**
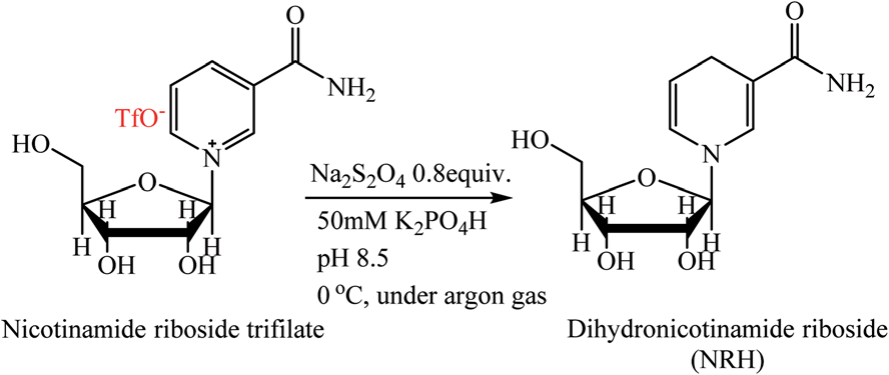
Synthesis of NRH from nicotinamide riboside triflate.^15^

There are very few methods for the preparation of NRH. This compound can be prepared from dihydronicotinamide mononucleotide (NMNH) by hydrolysis of the 5′-phosphate ester in the presence of alkaline phosphatase.^18^ This method is time-consuming and is not cost-effective because NMNH as a precursor must be enzymatically hydrolyzed from NADH. Another method for the synthesis of NRH is the reduction of NR in the presence of sodium dithionate (Na_2_S_2_O_4_) as a reducing agent.^15^ In this method, nicotinamide riboside triflate is reduced to NRH in an aqueous solution of sodium dithionate and potassium hydrogen phosphate (Scheme 2). Because the aqueous solution of Na_2_S_2_O_4_ is very unstable at ambient conditions, this reaction must be carried out at low temperature and under anaerobic, alkaline conditions. Furthermore, the crude product should be immediately purified with HPLC using a C18 resin because NRH is sensitive to both hydrolysis and oxidation at ambient conditions (discussed later in the Results and discussion section).

Although this method is suitable to synthesize a small amount of NRH, there are several drawbacks that prevent scaling this to the commercial production of NRH. The precursor, nicotinamide riboside triflate, is very expensive, is a very hygroscopic material, and must be stored at −20 °C under an inert atmosphere.^19^ Moreover, this compound is not food grade because of the presence of the triflate anion in the structure of nicotinamide riboside triflate. Therefore, a complete purification of NRH from the remaining NR (triflate) is required after the reduction.

**Scheme 3:**
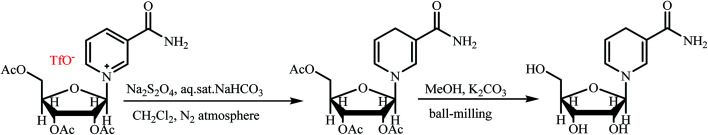
Indirect synthesis of NRH from triacetylated nicotinamide riboside triflate.^19,20^

Another method for the synthesis of NRH is the use of triacetylated nicotinamide riboside triflate instead of nicotinamide riboside triflate (Scheme 3).^19,20^ In the first step of this indirect procedure, triacetylated NR converts to triacetylated NRH with Na_2_S_2_O_4_. In the next step, the NRH is formed by methanolysis of triacetylated NRH while ball-milling. This method might be appropriate for scalable synthesis of NRH because it provides a good yield. However, the use of triacetylated nicotinamide riboside triflate, an expensive and non-food grade substance, may limit the applicability of this procedure.

It should be noted that most of the nicotinamide ribosides that have been used for the synthesis of NRH are the mixture of two anomeric α-and β-forms.^21^ However, only the β-anomer of NR represents bioactivity and medicinal properties.^21,22^ Among NR derivatives, only nicotinamide riboside chloride (NRCl) is commercially available as a dietary supplement.^21^ Therefore, using β-NRCl as a precursor to synthesize NRH represents a breakthrough for the commercialization of the NRH, a desired and valuable product.

**Scheme 4:**
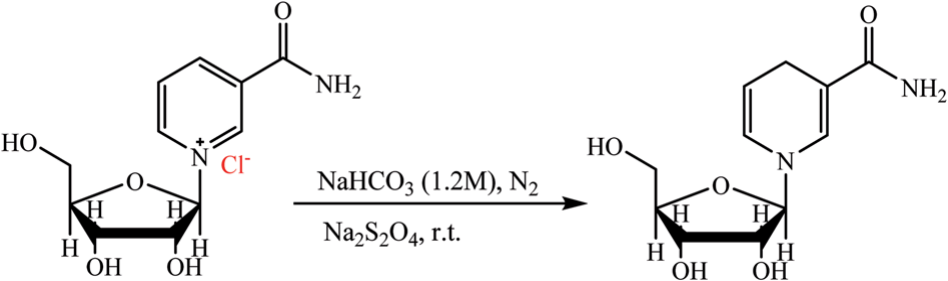
Synthesis of NRH from NRCl.

In the present study, we introduce a direct procedure for the scalable synthesis of NRH by using commercially available β-NRCl. The reaction was carried out in an aqueous solution of NaHCO_3_ and Na_2_S_2_O_4_ under a nitrogen atmosphere (Scheme 4). We developed a fast, column chromatography method for the purification of the NRH from the reaction mixture that results in 96% purity. Since one of the main applications of NRH is in supplemental beverages, we also investigated the effect of temperature, light, pH, and oxygen on the stability of NRH in aqueous solutions and compared these results to the stability of the NR in similar conditions.

## Experimental section

### Materials

NR chloride (beta form) was a gift from ChromaDex Company. Sodium dithionate was purchased from VWR, sodium hydrogen carbonate was purchased from Aldrich, silica gel (P60, 40–63 μm, 60 Å) was purchased from SiliCycle, and basic alumina (50–200 μm, 60 Å, pH 8) was purchased from Acros. Methanol (99.9%, Certified ACS, Fisher), acetone (99.8% Certified ACS, Fisher), sodium hydroxide (certified ACS, Fisher Chemical), hexanes (≥98.5%, GR ACS), ethyl acetate (EtOAc, >99.9%, certified ACS) and silica gel 60 F254 Coated Aluminum-Backed TLC Sheets were purchased from EMD Millipore (Billerica, MA, USA). Deuterated water and dimethyl sulfoxide (DMSO-d6, D, 99.9%) were purchased from Cambridge Isotope Laboratories, Inc.

### Characterization

A 500 NMR (Bruker) spectrometer was used to obtain the ^1^H and ^13^C-NMR spectra in deuterated water. Fourier transform infrared spectra (FTIR) were recorded on a Shimadzu IRAffinity-1S spectrophotometer by collecting 128 scans with a resolution of 8 cm^−1^. UV-vis spectra of the NR and NRH solutions were recorded on a Shimadzu UV-2600 spectrophotometer. Thermogravimetric analysis (TGA) thermograms were obtained in the range of 20–900 °C at a temperature rate of 10 °C min^−1^ under N_2_ flow, using a TA Q100 instrument. An Agilent 1200 LC System equipped with Binary SL Pump & Diode Array Detector and a Shodex RI-501 Refractive Index Detector (single channel) was used to perform the high-performance liquid chromatography (HPLC) measurements. Reversed-phase HPLC was performed on a Discovery C18 Column, 180 Å (pore size), 5 μm diameter, 250 × 4.6 mm in dimension. Ammonium acetate (20 mM) was used as the mobile phase with a flow rate of 1.0 mL min^−1^ over 30 or 45 min at 25 °C. All samples were filtrated using a 13 mm Nylon syringe filter with a 0.22 μm pore size before measurement. For LC-MS analysis, we used LC (Agilent 1100 series) coupled with a mass spectrometer. Reverse-phase chromatography was used with a Phenomenex Luna Omega (Phenomenex) LC column with the following specifications: 100 × 4.6 mm, 3 μm, polar C18, 100 Å pore size with a flow rate of 0.3 mL min^−1^. LC eluents include ammonium acetate 20 mM (solution A) and acetonitrile (solution B) using gradient elution (solution A : B composition change with time: 0 min: 95 : 5, 3 min: 95 : 5, 15 min: 85 : 15, 17 min: 90 : 10, and 20 min 95 : 5). The mass spectrometer (Finnigan LTQ mass spectrometer) was equipped with an electrospray interface (ESI) set in positive electrospray ionization mode for analyzing the NRH. The optimized parameters were a sheath gas flow rate at 20 arbitrary unit, spray voltage set at 4.00 kV, capillary temperature at 350 °C, capillary voltage at 41.0 V, and tube lens set at 125.0 V.

### Synthesis of 1,4-dihydronicotinamide riboside from β-nicotinamide riboside chloride

0.5 g of NRCl (1.72 mmol) and 20 mL of NaHCO_3_ solution (1.2 M) were added to a round bottom flask with a magnetic stir bar. This system was placed in an ice bath, and kept under nitrogen gas. Then, 0.80 g of sodium dithionate (4.60 mmol) was gradually added to the reaction mixture. After adding Na_2_S_2_O_4_, the flask was taken out of ice bath, and subsequently the reaction was carried out at room temperature for three extra hours. The reaction mixture was freeze-dried to obtain a yellow solid. Finally, the residue was purified by column chromatography using a mixture of basic alumina and silica with the weight ratio of 2 : 3 respectively, using methanol as eluent. The methanol was removed by rotary evaporator at room temperature to obtain a pale yellow, sticky solid that was next converted to a yellow powder (precipitate) by adding ethyl acetate. Finally, the isolated product was washed with *n*-hexane and dried under reduced pressure at room temperature to obtain pure NRH in 55% yield (0.24 g). ^1^H NMR (500 MHz, D_2_O), *δ* ppm: 7.19 (s, 1H), 6.14 (dd, *J*_1_ = 8.2 Hz, *J*_2_ = 1.5 Hz, 1H), 5.05–5.02 (m, 1H), 4.92 (d, *J* = 7 Hz, 1H), 4.24 (t, *J* = 5.5 Hz, 1H), 4.18–4.16 (m, 1H), 4.03–3.99 (m, 1H), 3.79 (dd, *J*_1_ = 12.5 Hz, *J*_2_ = 3.5 Hz, 1H), 3.73 (dd, *J*_1_ = 12.5 Hz, *J*_2_ = 5.0 Hz, 1H), 3.11 (s, 2H) (Fig. S1–S4, ESI†). ^13^C NMR (125 MHz, D_2_O), *δ* ppm: 173.03, 137.92, 125.32, 105.30, 101.05, 95.01, 83.62, 71.06, 70.24, 61.64, 22.09 (Fig. S5, ESI†). In addition, ^1^HNMR and ^13^C NMR spectra of purified NRH in CD_3_OD visuals are in the ESI section (Fig. S6–S9†) where MS: found *m*/*z* = 257.18 (M + 1). Calculated for C_11_H_17_N_2_O_5_ (M + 1): 257.11(Fig. S10, S11, ESI†). UV (*λ*_max_ in H_2_O): 338 nm (Fig. S12, ESI†).

### Stability study of the NRH and NR aqueous solutions

A stock solution of the freshly synthesized and purified NRH (10 000 ppm) was prepared in deoxygenated DI water. This stock solution was used for preparation of 1000 ppm NRH solutions for stability measurements. The effects of light, pH (buffer), temperature, and oxygen (as air) were investigated during a 60 day period of storage. The remaining NRH concentration was measured using HPLC. This procedure was also used for an NR stability measurement and the results were compared to the NRH samples at the same conditions.

## Results and discussion

**Scheme 5:**
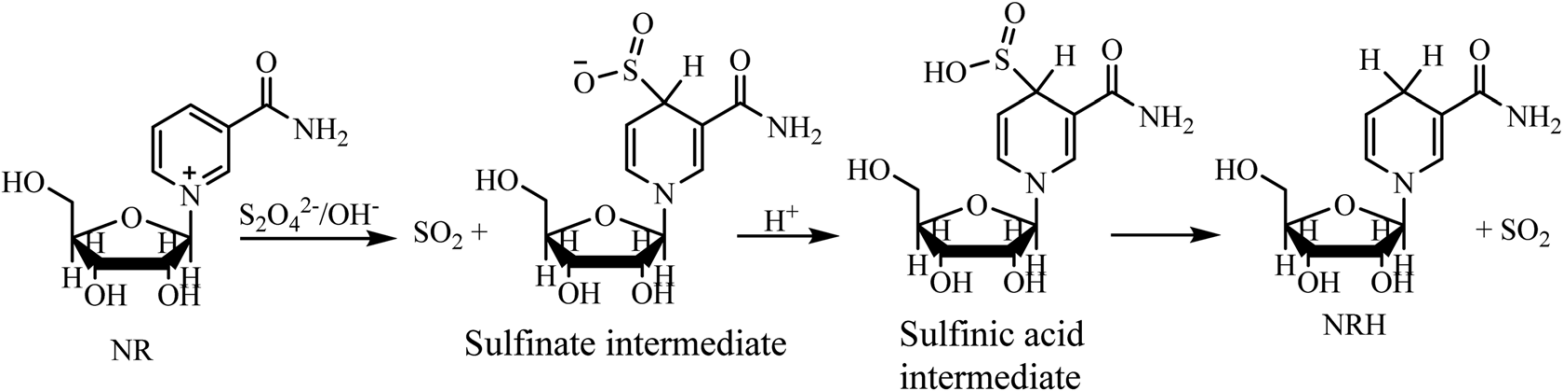
The mechanism of NRH synthesis from NR by using Na_2_S_2_O_4_.

One of the most significant parameters, and one which directly affects the NRH synthesis reaction and the NRH product, is the pH. Adjusting and maintaining a constant pH is important during the course of the reaction. This issue can be understood better by reviewing the mechanism of the reaction (Scheme 5).^23^ An aqueous solution of Na_2_S_2_O_4_ is unstable under aerobic conditions at pH = 7. Therefore, this reaction must be carried out under anaerobic and alkaline conditions.^24,25^ At first, the reaction between NR and S_2_O_4_^2−^ leads to the formation of a sulfinate intermediate, which is stable at basic conditions. By protonating this compound, its sulfinic acid derivative is formed. This sulfinic acid intermediate is unstable at ambient conditions and converts to NRH *via* releasing SO_2_. Therefore, it is important to find and establish a pH that will not only stabilize Na_2_S_2_O_4_ but will also protonate the sulfonate intermediate to produce the NRH. Moreover, because NRH has an *N*-glycoside bond in its structure, it is susceptible to hydrolysis.^15^ Consequently, it is necessary to adjust the pH and maintain it precisely during the whole course of the reaction so that NRH is not hydrolyzed.

In the first step, to decrease the hydrolysis of NRH during the course of the reaction, we set up the reduction reaction in a solvent-free environment. We used SiO_2_ as a solid support to increase the surface on which the reaction occurs. In this procedure, SiO_2_ (0.25 g), NRCl (0.1 g, 0.34 mmol), Na_2_S_2_O_4_ (0.2 g, 1.15 mmol), and NaHCO_3_ (0.25 g) were placed in a mortar and ground for 5 minutes to obtain a homogeneous powder. Then, 1 mL of DI water was dropwise added to the reaction mixture and the reaction was ground for 10 minutes at room temperature (Table 1, entry 1). It should be mentioned that without using the solid support the reaction mixture becomes sticky and is not easily ground. After grinding, the products were extracted by MeOH and the progress of the reaction was followed by TLC (thin layer chromatography). A trace amount of NRHwas detected; however, we could not isolate and purify the product for further characterization. We hypothesized that, in the absence of enough water, the sulfinate intermediate cannot be protonated to produce sulfinic acid intermediate and then NRH as the product (Scheme 5). To test this hypothesis, we repeated this procedure with an alternate final step. At the end, we transferred the reaction mixture to a round bottom flask with a magnetic stir bar, added 8 mL of NaHCO_3_ solution (1 M), and let the reaction continue on for 30 min at room temperature. Then, the reaction mixture was freeze-dried and the residue was purified by column chromatography in a mixture of basic alumina and silica, using methanol as the eluent. The isolated NRH was obtained with 15% yield (Table 1, entry 3). By changing the solid support (Al_2_O_3_ instead of SiO_2_), no remarkable improvement was observed in the yield of the product (Table 1, entries 2, 4).

**Table 1:** Synthesis of NRH from NRCl under different conditions

Entry	Reaction conditions	Base	Solid support	NRCl : Na_2_S_2_O_4_ (mol : mol)	Time	Yield^e^ (%)
1	Solvent-free^a^	NaHCO_3_ (0.25 g)	SiO_2_ (0.25 g)	1 : 3.4	15 min	0
2	Solvent-free^a^	NaHCO_3_ (0.25 g)	Al_2_O_3_ (0.25 g)	1 : 3.4	15 min	0
3	Solvent-free/solution^b^	NaHCO_3_ (0.25 g)	SiO_2_ (0.25 g)	1 : 3.4	15 + 30 min	15
4	Solvent-free/solution^b^	NaHCO_3_ (0.25 g)	Al_2_O_3_ (0.25 g)	1 : 3.4	15 + 30 min	17
5	Solution^c^	NaHCO_3_ (1.2 M)	—	1 : 2.0	3 h	42
6	Solution^c^	NaHCO_3_ (1.2 M)	—	1 : 2.5	3 h	50
7	Solution^c^	NaHCO_3_ (1.2 M)	—	1 : 2.7	3 h	55
8	Solution^c^	NaHCO_3_ (1.2 M)	—	1 : 3.0	3 h	54
9	Solution^d^	NaHCO_3_ (1.2 M) and Na_2_CO_3_ (0.2 g)	—	1 : 2.7	15 h	51^f^

a NRCl (0.1 g), Na_2_S_2_O_4_ (0.2 g), base and solid support were placed in a mortar and ground.

b NRCl (0.1 g), Na_2_S_2_O_4_ (0.2 g), base and solid support were placed in a mortar and ground for 15 min, then all the reaction mixture were added to 8 mL of NaHCO_3_ (1 M) solution and stirred for 30 min.

c The reaction of NRCl (0.5 g) and Na_2_S_2_O_4_ (0.8 g) was carried out in 20 mL of NaHCO_3_ (1.2 M) solution under N_2_ atmosphere at pH 8.1.

d The reaction of NRCl (0.5 g) and Na_2_S_2_O_4_ (0.8 g) was carried out in a solution of 20 mL of NaHCO_3_ (1.2 M) and 0.2 g of Na_2_CO_3_ under N_2_ atmosphere at pH 8.5.

e The yields refer to the isolated pure products.

f The product contains 12% of dihydronicotinamide impurity based on ^1^H NMR.

The ability to work under ambient conditions is an advantage of this procedure, but low yield of the product is a serious drawback. So, in search of a means for producing a high yield, we decided to follow the reaction in aqueous solution under anaerobic conditions (Table 1, entries 5–9).

**Fig. 1:**
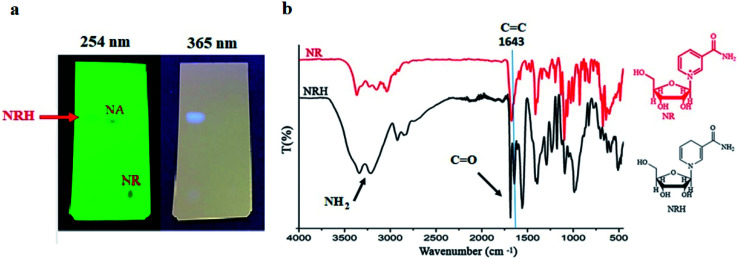
(a) The TLC of synthesized NRH at pH 8.1 after purification compared to NR and NA. (b) FT-IR of purified NRH and pristine NR.

The minimum pH to stabilize the aqueous solution of Na_2_S_2_O_4_, without decreasing its activity as a reducing agent, is between 8.0–8.5.^15,19^ Initially, we set up the reaction at pH 8.1 by employing a solution of NaHCO_3_ (1.2 M). Next, NRCl was dissolved in this solution and Na_2_S_2_O_4_ was gradually added to the reaction mixture, under a nitrogen atmosphere at 0 °C. By adding Na_2_S_2_O_4_ to the solution, and throughout its oxidation, the pH decreased. However, the sodium bicarbonate solution with the concentration of 1.2 M was enough to keep the pH constant during the course of the reaction.^26^ After adding Na_2_S_2_O_4_, the reaction was carried out at room temperature for 3 hours. Then, the mixture was freeze-dried to obtain a yellow solid. Finally, the residue was purified by column chromatography with a mixture of basic alumina and silica (methanol as eluent) to obtain the pure NRH product in 55% yield (Table 1, entry 7). The optimized molar ratio between NRCl and Na_2_S_2_O_4_ was 1 to 2.7 and an increase in the amount of sodium dithionate did not improve the product yield (Table 1, entry 8). After purification of the crude product, we conducted thin layer chromatography (TLC) with the purified NRH using methanol as a solvent and compared with the pristine NRCl and nicotinamide (NA) (Fig. 1a). All compounds were active at a wavelength of 254 nm. However, by switching the wavelength to 365 nm, we found that only NRH fluoresced. It has been reported that NRH is strongly fluorescent around 340 nm.^15^ Because the *R*_f_ values of NRH and NA were almost the same, the fluorescence property of NRH helped to identify the exact position of this compound during TLC. As shown in Fig. 1a, the purified NRH shows high purity on the TLC plate.

The FT-IR spectrum of the synthesized and purified NRH was studied and compared to the spectrum of the pristine NRCl (Fig. 1b). In the FT-IR spectrum of NRH, the existence of a specific peak at 1643 cm^−1^ can be attributed to the stretching vibration of C=C band,^27^ and confirms the reduction of the NR pyridinium ring. Two peaks at 3340 and 3209 cm^−1^ refer to asymmetric and symmetric stretching bands of NH_2_ group^27^ in the structure of NRH which implies that the amide group is intact during the course of the reaction. A sharp peak at 1685 cm^−1^ indicates the stretching C=O band of the amide group.^27^ Two peaks at 2920 and 2840 cm^−1^ confirm the stretching vibration of aliphatic C–H in both the ribose and the dihydronicotinamide rings of the NRH structure. The presence of hydroxyl groups in the structure of NRH is confirmed with a broad peak that appears in the range of 3500 to 3000 cm^−1^.^27^

**Fig. 2:**
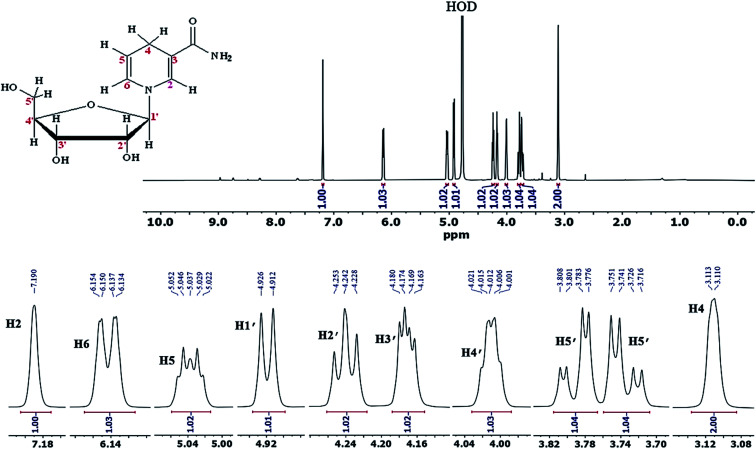
^1^HNMR of purified NRH in D_2_O.

In order to further ensure the successful synthesis of NRH and its purity, we also took ^1^HNMR and ^13^CNMR spectra of the purified NRH in deuterated water. The existence of eleven protons that are not exchangeable with D_2_O completely confirms the structure of synthesized NRH (Fig. 2). The chemical shift of each proton and the corresponding coupling constants are in agreement with the ones previously reported in the scientific literature.^19^ The ^13^CNMR and more details are given in ESI (Fig. S5).†

**Fig. 3:**
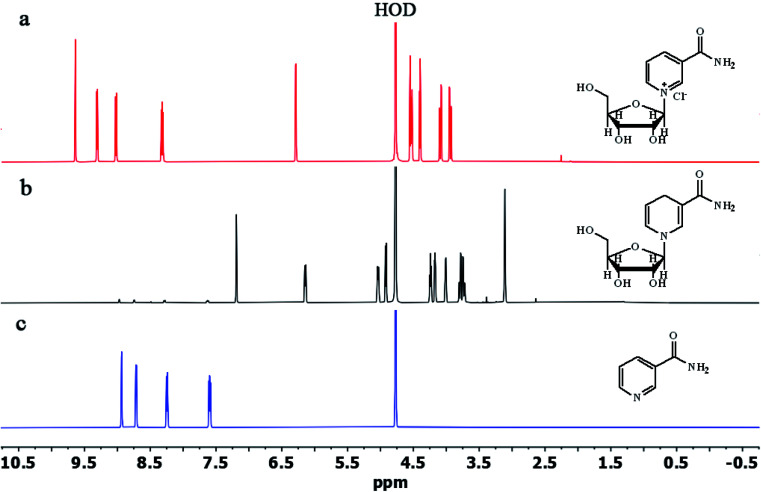
The ^1^H NMR spectra of (a) NRCl, (b) NRH and (c) NA in D_2_O.

To study the efficiency of the column used for the purification of NRH, we compared the ^1^HNMR of purified NRH with the ^1^HNMR of pure NRCl and NA (Fig. 3). The major impurity in the reaction mixture was unreacted NR and, after purification, we observed no specific NR peak in the ^1^HNMR spectrum of purified NRH. By comparing the NRH spectrum with the NA spectrum, we found that there were only trace peaks, thus indicating NA in the ^1^HNMR of purified NRH. This signifies that only a trace amount of NR hydrolyzes during the course of the reaction to form NA. We also used reverse phase chromatography HPLC to further confirm the purity of the NRH sample (Fig. S13†). The HPLC findings agreed with ^1^HNMR data and showed both that the purity of NRH was 96% and the impurity of NA was 4%. Interestingly, no NR was detected with HPLC, although NR was present with ^1^HNMR. This means that column chromatography on a mixture of basic alumina and silica is very efficient and able to completely purify the NRH product from the remaining NRCl.

Next we tried to purify crude NRH, using column chromatography on silica and using methanol as the eluent. The results showed that column chromatography on silica alone was not effective in separating NR from NRH. Basic alumina, as a polar surface with many hydroxyl groups on it, can, to a moderate degree, separate NR from NRH (by difference of their polarities). Moreover, because basic alumina has negative charges on its surface and NR has a positive charge, it can act as a cationic exchange resin to immobilize NR on its surface. We believe this is the main reason why alumina is a better stationary phase compared to silica for the purification of the NRH. However, we discovered that during the purification of NRH in the column chromatography, basic alumina would clog and result in a very slow flow rate that led to the degradation of NRH. We took steps to easily solved this problem, by mixing basic alumina with silica with the weight ratio of 2 : 3 respectively. Thus, for the first time, we could quantitatively purify NRH in a preparative mode (without using HPLC) using a common column chromatography method on a mixture of basic alumina and silica, while using methanol as the eluent.

**Fig. 4:**
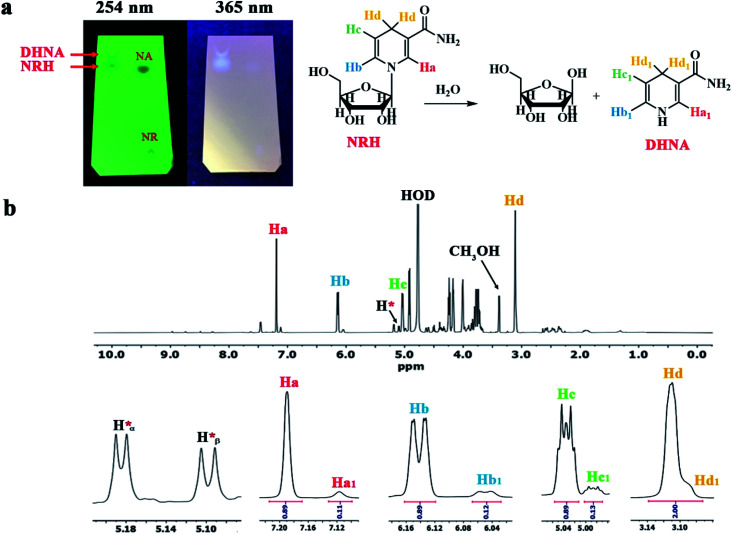
(a) The TLC of synthesized NRH at pH 8.5 after purification compared to NRCl and NA. (b) The ^1^HNMR of synthesized NRH at pH 8.5 after purification.

Next we set up the reduction reaction of NRCl at pH 8.5 by employing a solution of NaHCO_3_ and Na_2_CO_3_ (Table 1, entry 9). The NRCl was dissolved in this solution, then Na_2_S_2_O_4_ was gradually added to the reaction mixture under a nitrogen atmosphere at 0 °C. After the addition of Na_2_S_2_O_4_, the reaction was carried out at room temperature for 15 hours. Upon completion of the reaction (followed by TLC), the mixture was freeze-dried to obtain a yellow solid. Finally, the residue was purified by short column chromatography on basic alumina using methanol as the eluent. As a qualitative test, we conducted TLC from this purified NRH and compared with the NR and NA using methanol as a solvent (Fig. 4a). The results showed that the NRH obtained was not pure after column chromatography. At this point we observed that the new impurity was positioned only slightly above the NRH on the TLC. Our observation helped to explain why this contamination cannot be separated from the NRH by common column chromatography. Interestingly, the fluorescence property of this new impurity is similar to NRH, causing both of them to fluoresce at 365 nm on TLC. By studying the literature, we found that it was likely 1,4-dihydronicotinamide (DHNA) formed by hydrolysis of the NRH during the course of the reaction.^15^

For further evidence, we took ^1^HNMR of this sample in D_2_O (Fig. 4b). The obtained results indicated that there was no residual NRCl in this sample. However, the presence of four peaks with low intensity between 7.5–9 ppm confirmed the existence of a trace amount of NA in the purified NRH. As shown in Fig. 4b, four main peaks at 7.19, 6.14, 5.03 and 3.11 ppm strongly confirm a 1,4-dihydronicotinamide ring in the structure of NRH. At the right side of each of the main peaks, there is a peak with lower intensity and a shape is similar to the corresponding main peak. The chemical shifts of these peaks are 7.12, 6.05 and 4.98 ppm, respectively. The integral of each peak is around 0.12 and the integral of each main peak is 0.89.

These results demonstrate that around 12% of DHNA exist in the purified NRH. In other words, this means that by increasing the pH, the reaction rate decreases and the NRH is hydrolyzed under the reaction conditions within 15 hours. It is clear that by hydrolyzing NRH, D-ribose and DHNA are formed simultaneously. As shown in Fig. 4b, the corresponding impurity peaks at 5.19 and 5.10 ppm are attributed to α and β anomeric protons of D-ribose, respectively.^28^ The other impurity peaks of ribose appear between 3.65 and 4.65 ppm.

By increasing the pH from 8.1 to 8.5, the reaction rate obviously decreases and, subsequently, there is an increased possibility of side reactions, such as hydrolysis of both NR and NRH.

These observations are in accordance with the reaction mechanism of the reduction of NR to NRH, as seen in Scheme 5. In this mechanism, the reaction involves a sulfinate intermediate which is stable in alkaline conditions and, consequently, it is not easily protonated to produce the NRH. In the process of NRH synthesis, thiosulfate and bisulfite anions are formed as the products of hydrolysis and oxidation of dithionate.^24^ These anions, with high nucleophilicity,^29^ may attack the carbon 1′ in the structure of NRH and substitute instead of DHNA. This would indirectly increase the rate of NRH hydrolysis. By increasing the pH, the possibility of NRH hydrolysis decreases,^15^ while simultaneously the existence of some anions, which act as the nucleophile, can increase the NRH hydrolysis. The hydrolysis of NRH by these anions is more severe and increases the reaction time of NRH synthesis. Therefore, as the most important parameter, the pH of the reaction must be adjusted to minimize the formation of NA and DHNA to decreasing the reaction time. This is a very significant factor because the *R*_f_ values of these by-products are close to the *R*_f_ values of NRH; therefore, they cannot be separated from the NRH product by common column chromatography (Fig. 4a). In the present work, we found that the optimized pH for the synthesis of NRH from NR was 8.1; at this pH (8.1) we did not observe any DHNA and the amount of NA was negligible.

**Fig. 5:**
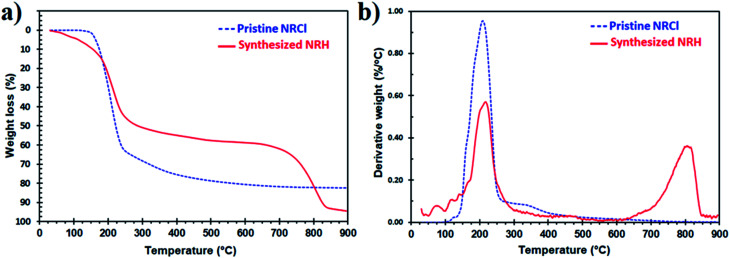
TGA thermograms of the NRH compared to the NRCl in the range of 25 to 900 °C under N_2_ atmosphere. (a) Weight loss *vs.* temperature, (b) derivative weight loss *vs.* temperature.

Thermal stability is very important for drugs and supplements that have the potential for use in food due to the high temperatures that are often needed for industrial processing. To study the thermal stability of the NRH, we performed TGA for the pure NRH from 25 to 900 °C and the results were compared to the pristine NRCl. Fig. 5 shows the TGA thermograms in the range of 25 to 900 °C for NRH and NRCl. As can be seen in Fig. 5a and b, the NRH is clearly more thermally stable than the NRCl. While both NRH and NR show their greatest weight loss at around 218 °C and 910 °C, respectively, NRH shows only around 50% weight loss at this temperature, while NRCl shows around 65% weight loss at this temperature.^30^ NRH shows another distinctive weight loss with a peak at around 800 °C which contributes to 30% weight loss. These results confirm that the NRH is more thermally stable compared to the NRCl under a nitrogen atmosphere. It may be due to the existence of chloride ions in the structure of NRCl, that can leave in the form of HCl when the temperature increases.

### Study of the stability of NRH in different conditions

**Scheme 6:**
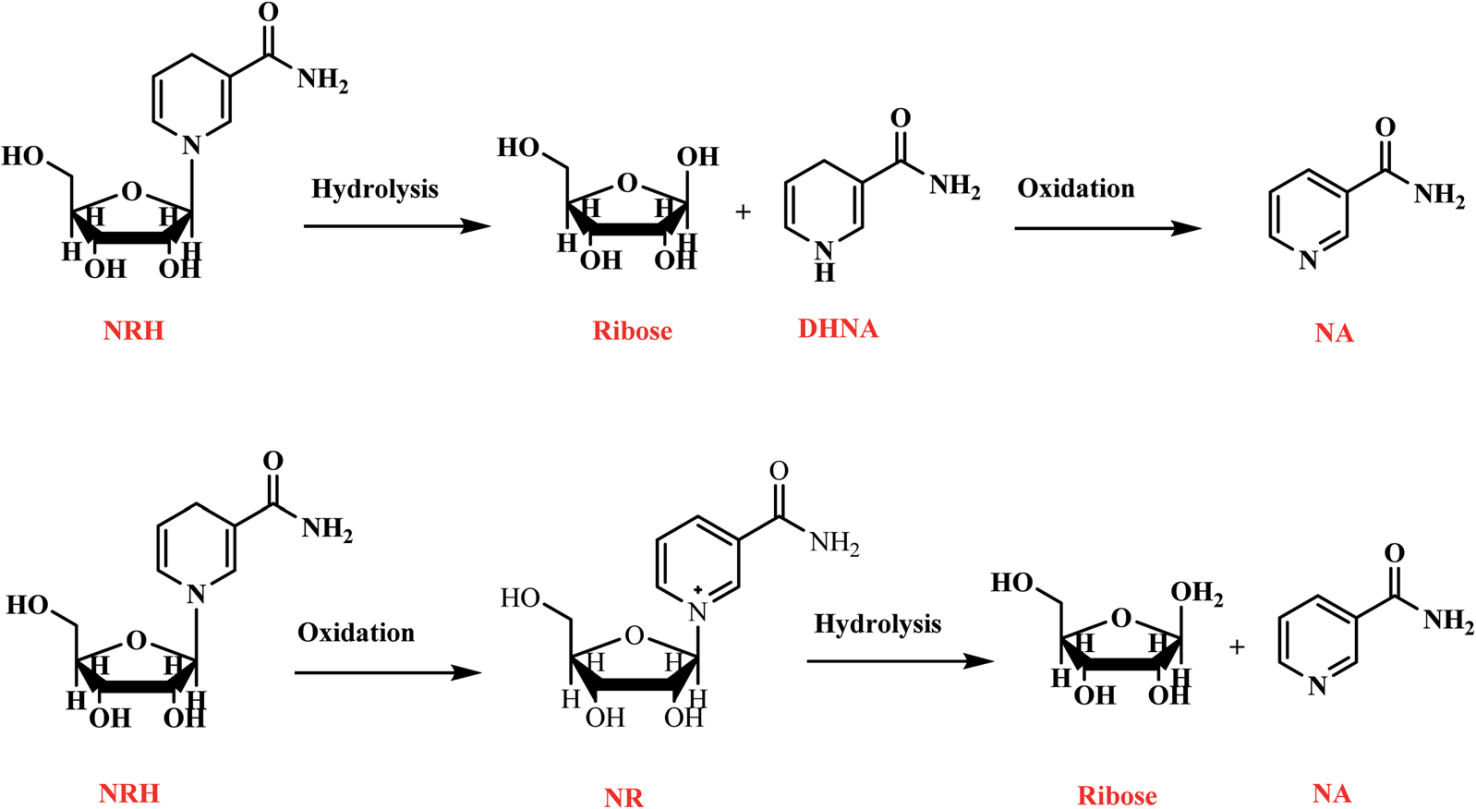
The degradation products of NRH including hydrolysis and oxidation.

NRH is an unstable, sensitive molecule due to its *N*-glycosidic bond and can undergo degradation, hydrolysis, and oxidation during exposure to high temperatures, nucleophiles, and oxygen. Some of the major products of these degradation reactions for NRH are shown in Scheme 6. Studying the NRH degradation under different storage conditions is important for developing new supplements or potential beverage products. Here, we study the effect of light, temperature, oxygen, and pH on the degradation rate of the freshly synthesized and purified NRH in an aqueous solution during a period of 60 days.

#### Effect of oxygen and light

**Fig. 6:**
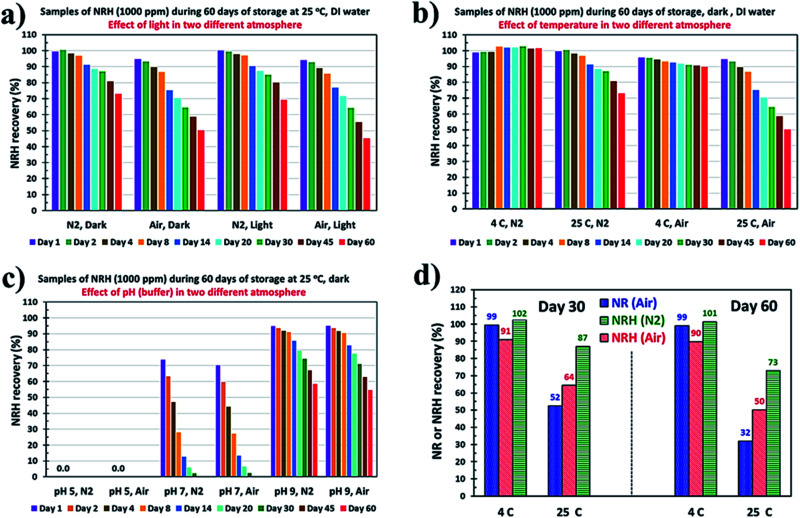
NRH degradation kinetic study. (a) NRH recovery (%) in aqueous samples during the 60 days of storage in the dark/light and in air/under N_2_, (b) recovery of the NRH in aqueous solutions that are being kept at 4 °C and 25 °C during 60 days of storage in air/under N_2_, (c) the effect of pHs 5, 7, and 9 buffers on the stability of the NRH during 60 days storage at 25 °C in air and under N_2_, (d) NR and NRH recovery (%) in aqueous samples during the 30 days, and 60 days of storage at 4 and 25 °C.

The oxidative sensitivity of the NRH, as a dihydronicotinamide derivative, is well known as the reduced form of the NR and, therefore, has a high oxidation potential.^31^ To study the effect of air, N_2_ atmosphere, as well as ambient light, on the stability of NRH in an aqueous solution, we prepared solutions of freshly purified NRH (by column chromatography) in DI water. We monitored the NRH concentration over the course of 60 days by HPLC. Fig. 6a shows the NRH recovery (%) during the 60 days of storage based on the HPLC results both for samples in the dark/light and in air/under N_2_.

From the result shown in Fig. 6a, it is obvious that, while the effect of light is negligible on the stability of NRH compared to the samples that have been kept at dark, oxygen (as air) has a dramatic effect on the stability of the NRH. Samples that have been kept at 25 °C in DI water in darkness show faster degradation reaching 50% in 60 days under air atmosphere. By contrast, there is around 27% degradation observed for the sample at the same conditions that have been kept under a N_2_ atmosphere. These findings demonstrate that NRH is susceptible to oxidation in the presence of air.

#### Effect of temperature

Temperature can have a profound effect on the stability of molecules with an *N*-glycosidic bond.^15,32^ The NR molecule degradation is temperature dependent following a first order kinetic rule.^33^ This is most likely due to the susceptibility of the *N*-glycosidic bond dissociation because nicotinamide (NA) is a good leaving group.^33^ However, the leaving group for NRH is dihydronicotinamide (DHNA) and NRH is not ionic (nitrogen in the ring does not have a positive charge). Therefore, we might anticipate that the NRH should be more stable compared to the NR molecule in terms of spontaneous dissociation of the *N*-glycosidic bond due to the temperature. However, the oxidative degradation of the NRH also plays an important role for its stability as we showed in previous section.

We study the stability of the NRH at two different temperatures both in air and under a N_2_ blanket. Fig. 6b shows the results of the recovery of the NRH in aqueous solutions kept at 4 °C and 25 °C during 60 days of storage. Fig. S14† shows the corresponding HPLC chromatograms of some of the samples in 60 days of storage. These results confirm the faster degradation of the NRH at 25 °C compared to 4 °C, and also show the accelerated effect of air on the oxidative degradation of NRH at 25 °C. Samples that have been kept at 4 °C under a N_2_ blanket do not show any detectable degradation after 60 days of storage, while samples at 4 °C in air display around 10% degradation in 60 days. For the samples at 25 °C, the degradation is around 27% under a N_2_ atmosphere and around 50% in air after 60 days of storage. Pure NRH (as a powder) was stable in a refrigerator, in a sealed tube, for a few months without any degradation.

#### Effect of pH (buffer)

Dissociative degradation of the NR molecule is pH independent.^32,33^ The NRH molecule showed relatively good stability in a basic medium (as we also emphasize in the Results and discussion) and rapid degradation under acidic conditions during a 10 h monitoring period.^15^ We investigated the effect of pH 5, 7, and 9, made using citrate buffer, ammonium acetate buffer, and carbonate buffer, respectively, on the stability of the NRH in the aqueous solutions kept at 25 °C both in air and under N_2_, as well as in darkness, for a period of 60 days (Fig. 6c and S15†). Our results from Fig. 6c confirm the complete degradation of NRH in less than one day at pH 5, while samples prepared in the ammonium acetate buffer at pH 7 showed a linear decrease of NRH concentration from around 70% in day one to around 2% in day 30. However, samples prepared in the carbonate buffer at pH 9 showed fair stability both in air (around 45% degradation measured after 60 days) and under N_2_ (around 42% degradation measured after 60 days), which agrees with the short term stability data from others.^15^

Comparing Fig. 6c (25 °C, pH 7 air and N_2_) with Fig. 6b (25 °C, DI water), it is clear that the rapid degradation of the NRH at pH 7 is due to the ammonium acetate salt used to make the buffer. The presence of some anions, such as chloride and acetate, accelerate the oxidation of NRH to NR because the existence of an anion as a counter ion of NR is necessary in this oxidation process.^21^ Moreover, the acetate ion may act as a nucleophile and accelerate the NRH hydrolysis.^21^ By consuming the acetate anion, the ratio of ammonium to acetate increases and, by hydrolysis of this extra quantity of ammonium, the solution gradually becomes acidic and the NRH hydrolysis rate increases. Therefore, it can be stated that there is a synergistic effect for the degradation of the NRH while at pH 7.0 using a buffer produced with ammonium acetate.

The kinetic graphs for the NRH degradation rate are based on the HPLC data collected during the 60 days of storage for samples with rather fast degradation rates. Fig. S16† shows the kinetic graphs and the first order degradation rates for NRH samples stored at pH 7.0, at 25 °C, under N_2_ and air (Fig. S16a, b†), samples stored at pH 9.0, at 25 °C, under N_2_ and air (Fig. S16c, d†), and for NRH samples that have been kept in DI water, at 25 °C, under N_2_ and air (Fig. S16e, f†). The degradation rate for samples that have been kept at 25 °C in DI water under air is 1.27 × 10^−7^ s^−1^, which corresponds to a half-life of 63 days. By comparison, for the NRH samples that have been kept at 25 °C in DI water under N_2_ the degradation rate is 5.90 × 10^−8^ s^−1^, which correspond to a half-life of 136 days (Fig. S16†).

### Comparison of the stability of NRH *vs.* NR in aqueous solution

NRH is the reduced form of NR and has a much higher biological activity.^15,17^ However, it is essential to compare the stability of NR to NRH. If the NRH is not as stable as NR, the latter would be useful for more applications. Here, we compare the stability of both NR and NRH in aqueous solutions. Since NR stability in aqueous solutions is pH independent,^33,34^ we studied the NR and NRH stability in DI water at 4 °C and 25 °C for a period of 60 days. Fig. 6d compares the results of this study for 30 and 60 days.

It is clear from the results of Fig. 6d that, at 4 °C, the NR in an aqueous solution is very stable with no detectable degradation after 60 days. However, NRH in an aqueous solution stored in air shows 9% and 10% degradation after 30 and 60 days, respectively, while NRH in an aqueous solution stored under a N_2_ blanket is fairly stable with no detectable degradation after 60 days of storage. On the other hand, the NR solution that has been kept at 25 °C (representing the ambient temperature) shows 48% and 68% degradation after 30 and 60 days of storage, while this degradation is 36% and 50% for NRH solutions that have been kept under air after 30 and 60 days and 13% and 27% for and NRH solutions that have been kept under N_2_ after 30 and 60 days, respectively. This clearly shows that the thermal degradation is more severe for NR than NRH in aqueous solutions, and storage of NRH away from air improves its stability.

## Conclusions

A convenient, efficient, and scalable procedure for the synthesis and purification of NRH from the commercially available NRCl was developed. In the present method, sodium dithionate was used as a reducing agent under different conditions. While the aerobic solid phase synthesis could not provide high yields, the anaerobic synthesis in a solution of sodium bicarbonate (1.2 M), at pH = 8.1, reached around 55% yield after a 3 h reaction time. For the first time, the purification process was done by using a common column chromatography with basic alumina. We found the effect of the reaction's pH was very critical and is optimized at 8.1. Maintaining this pH is essential throughout the entire reaction. We showed that an increase from pH = 8.1 to pH = 8.5 led to an increase in the reaction time and some significant side reactions including hydrolysis of the NRH to ribose and dihydronicotinamide. NMR, FTIR, LC-MS, UV-vis, and HPLC confirmed the structure as well as the high purity of the NRH synthesized by this method. To investigate the effect of temperature, pH, light, and oxygen (air) on the NRH degradation in aqueous solutions, we performed a long-term stability study. Results showed that NRH was oxidized in the presence of oxygen, and it hydrolyzed quickly in acidic conditions. The degradation rate was lower under a N_2_ atmosphere, at lower temperatures, and in a basic pH.

## Conflicts of interest

The authors declare no competing financial interest.

## Supplementary Material

RA-011-D1RA02062E-s001
