# Supplementary material for: Dihydronicotinamide riboside: synthesis from nicotinamide riboside chloride, purification and stability studies
Source: RSC Adv. 2021 Jun 14;11(34):21036–47. doi: 10.1039/d1ra02062e (PMC9034155; doi:10.1039/d1ra02062e)
Supplement: RA-011-D1RA02062E-s001 [file RA-011-D1RA02062E-s001.pdf]

Electronic Supplementary Material (ESI) for RSC Advances.
This journal is © The Royal Society of Chemistry 2021

Supporting Information

Dihydronicotinamide riboside: synthesis from nicotinamide riboside chloride, purification and stability studies

Amin Zarei[a]†, Leila Khazdooz[a]†, Mojtaba Enayati[a], Sara Madarshahian[a], Timothy J. Wooster[b], Gerhard Ufheil[c], Alireza Abbaspourrad[a]*

[a]Department of Food Science, College of Agriculture and Life Sciences, Cornell University, Ithaca 14853, NY, USA

[b]Institute of Material Science, Nestle Research, CH-1000 Lausanne 26, Switzerland

[c]Nestle Product Technology Center Bridgewater, Nestle Nutrition R&D Inc., Bridgewater, NJ 08807, USA

†These authors contributed equally to this work.

Corresponding Author; Email: Alireza@cornell.edu (A.A.)

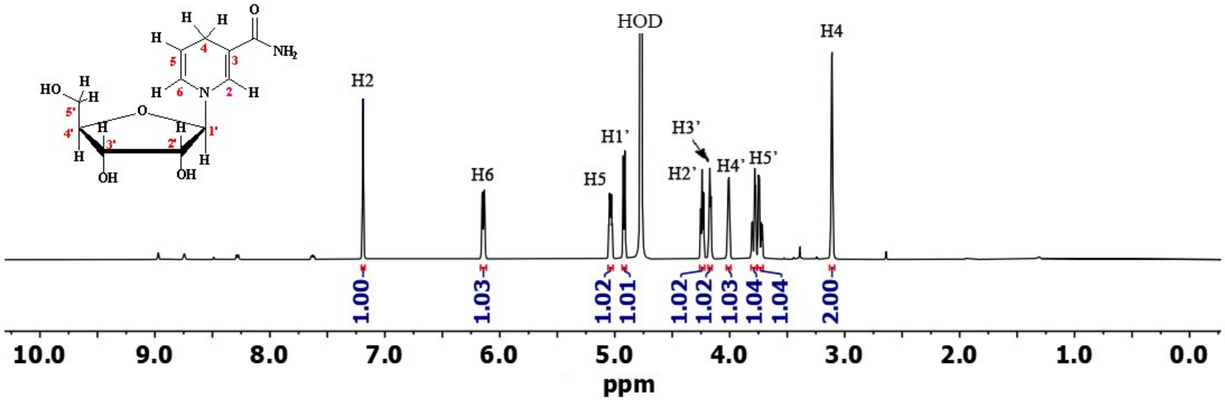

Figure S1. ^{1}H NMR Spectrum of NRH in D_2O.

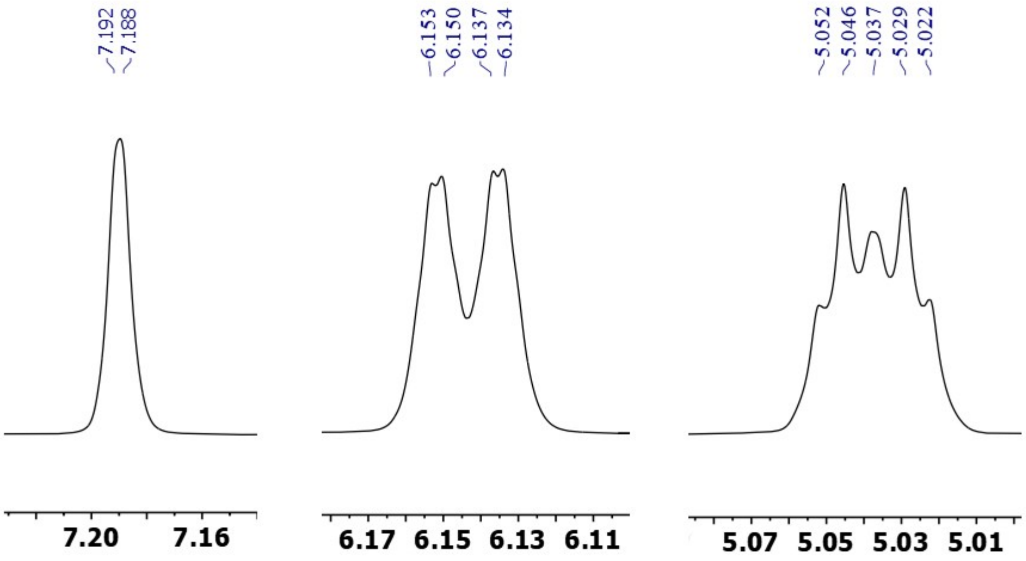

Figure S2. ^{1}H NMR Spectrum of NRH in D_2O. Expanded between 5.00-7.20 ppm.

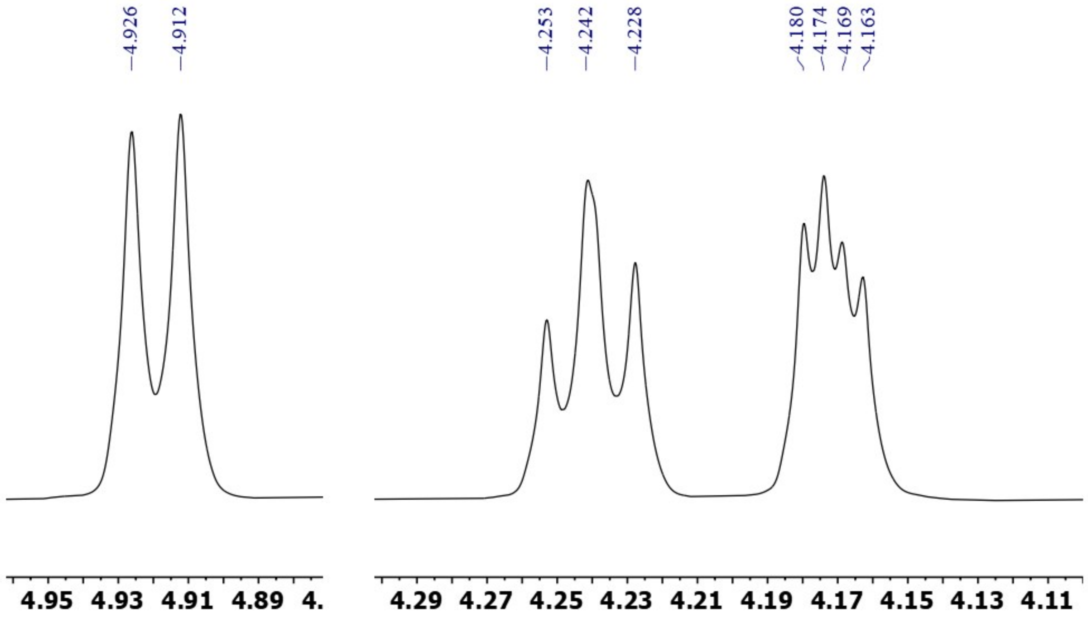

Figure S3. ^{1}H NMR Spectrum of NRH in D_2O. Expanded between 4.11-4.95 ppm.

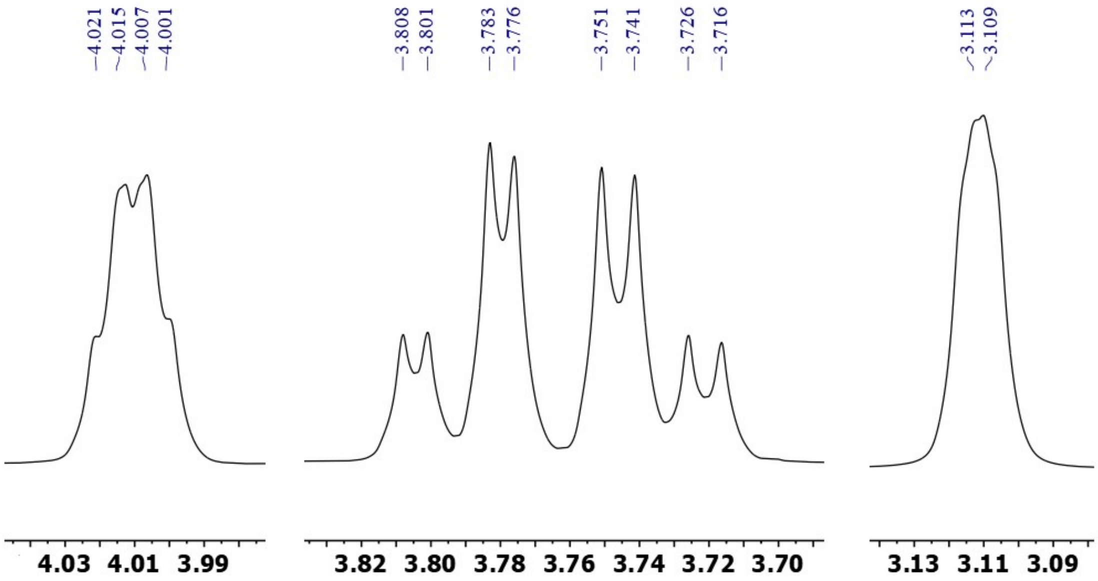

Figure S4. ^{1}H NMR Spectrum of NRH in D_2O. Expanded between 3.09-4.03 ppm.

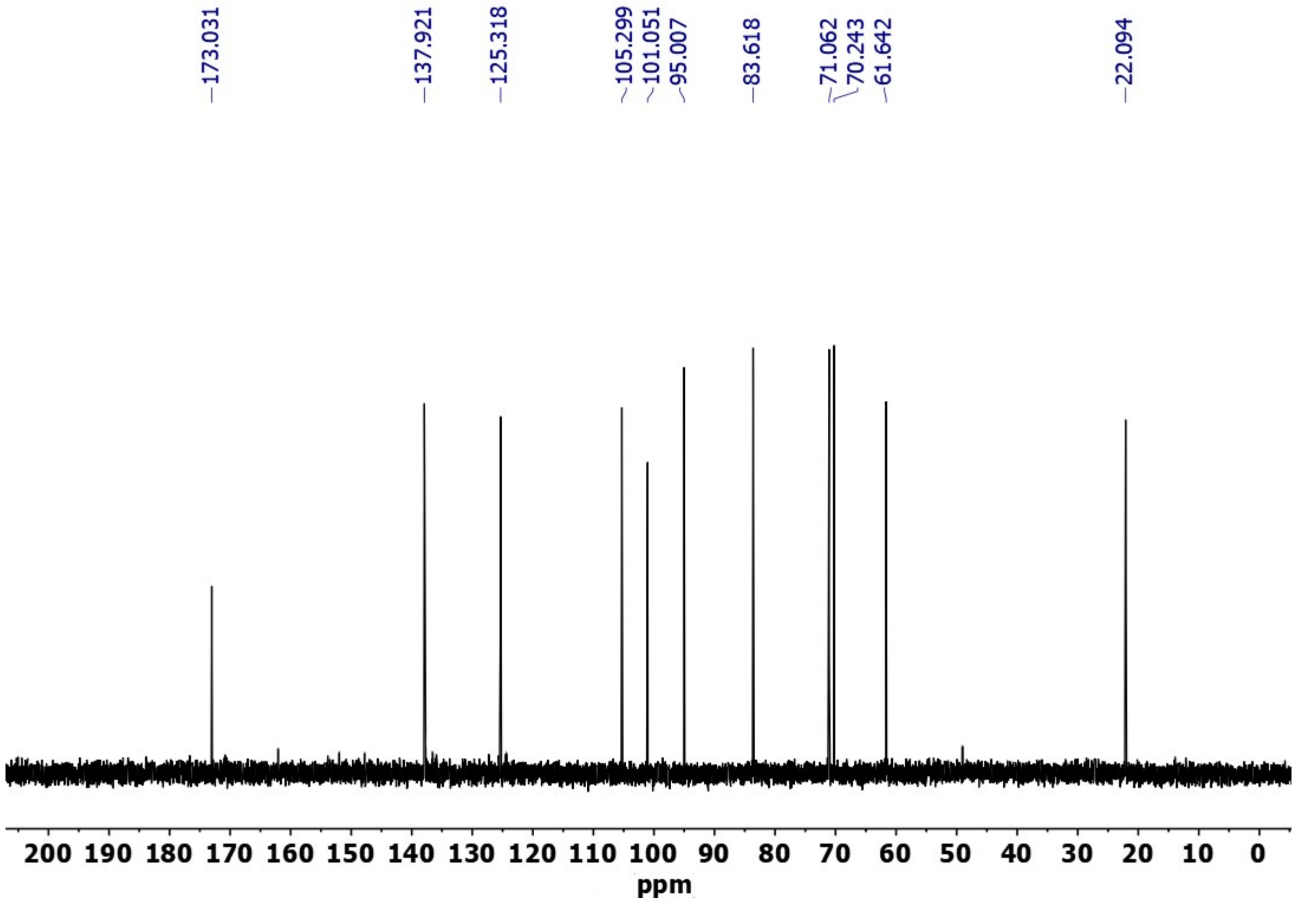

Figure S5. ^{13}C NMR Spectrum of NRH in D_2O.

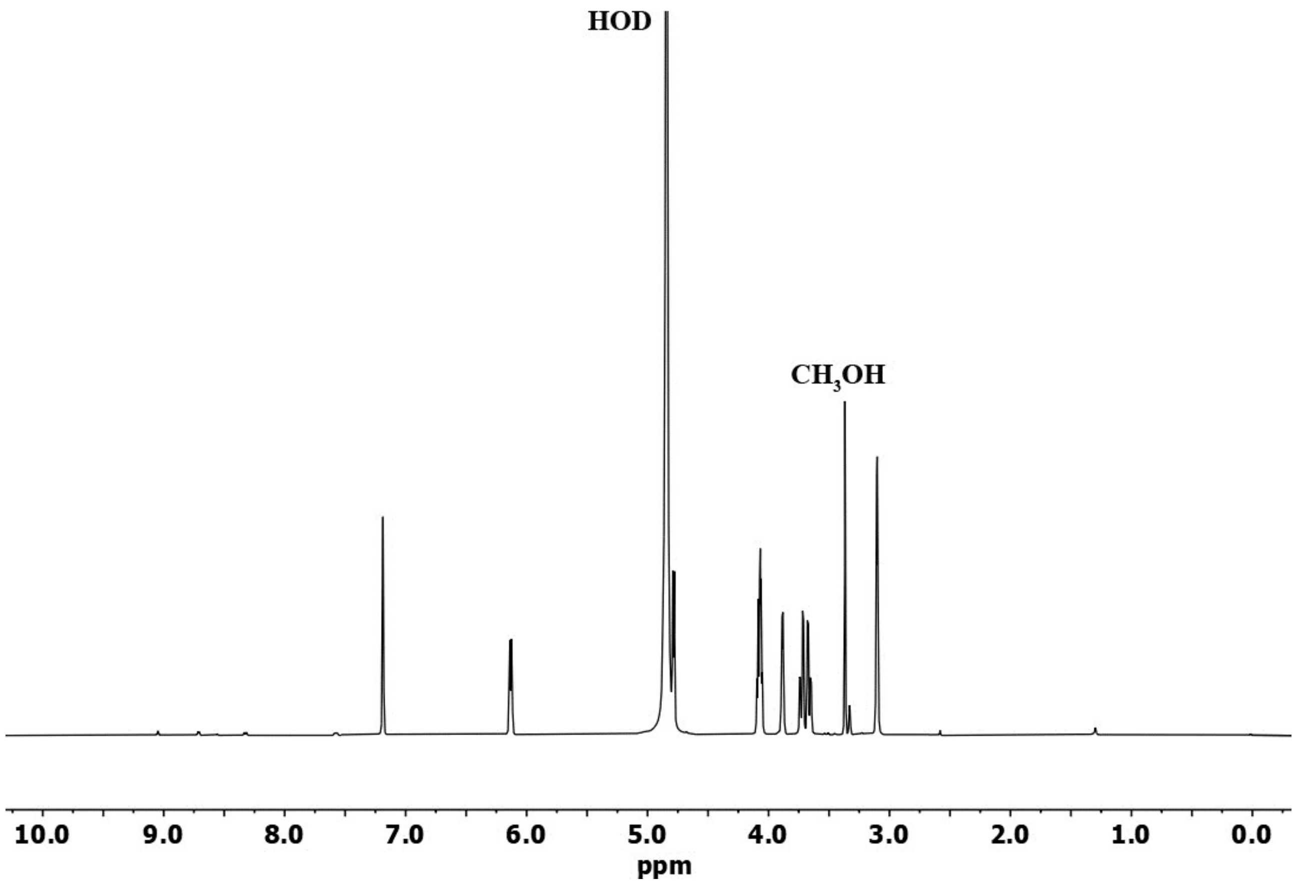

Figure S6. ^{1}H NMR Spectrum of NRH in CD_3OD.

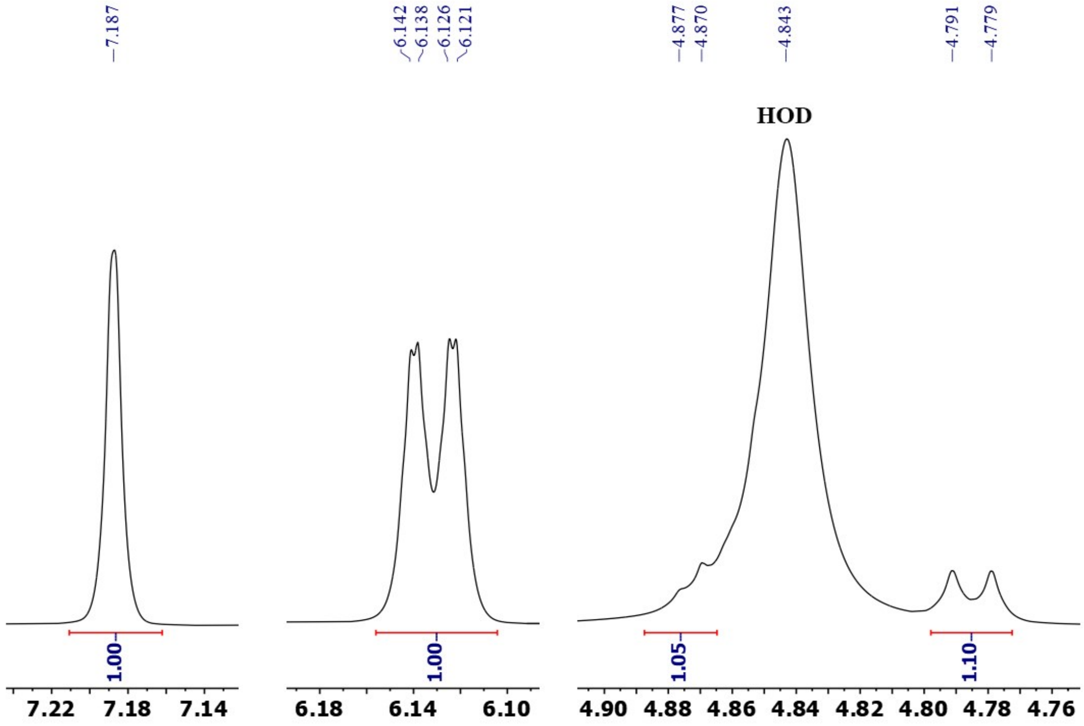

Figure S7. ^{1}H NMR Spectrum of NRH in CD_3OD. Expanded between 4.76-7.22 ppm.

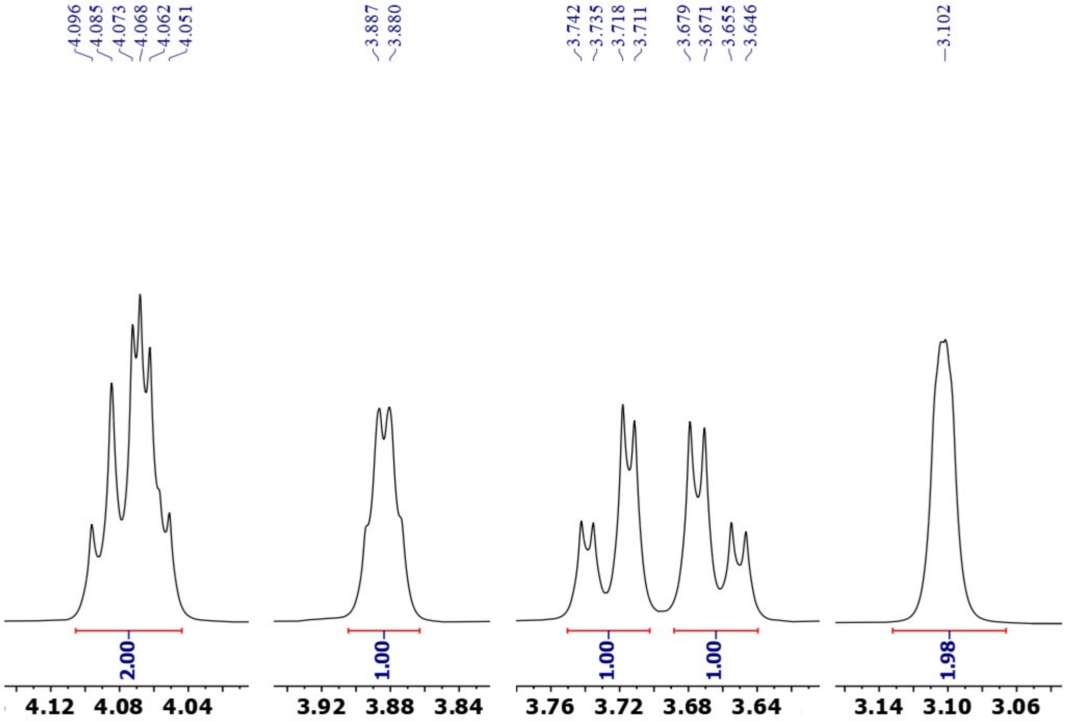

Figure S8. ^{1}H NMR Spectrum of NRH in CD_3OD. Expanded between 3.06-4.12 ppm.

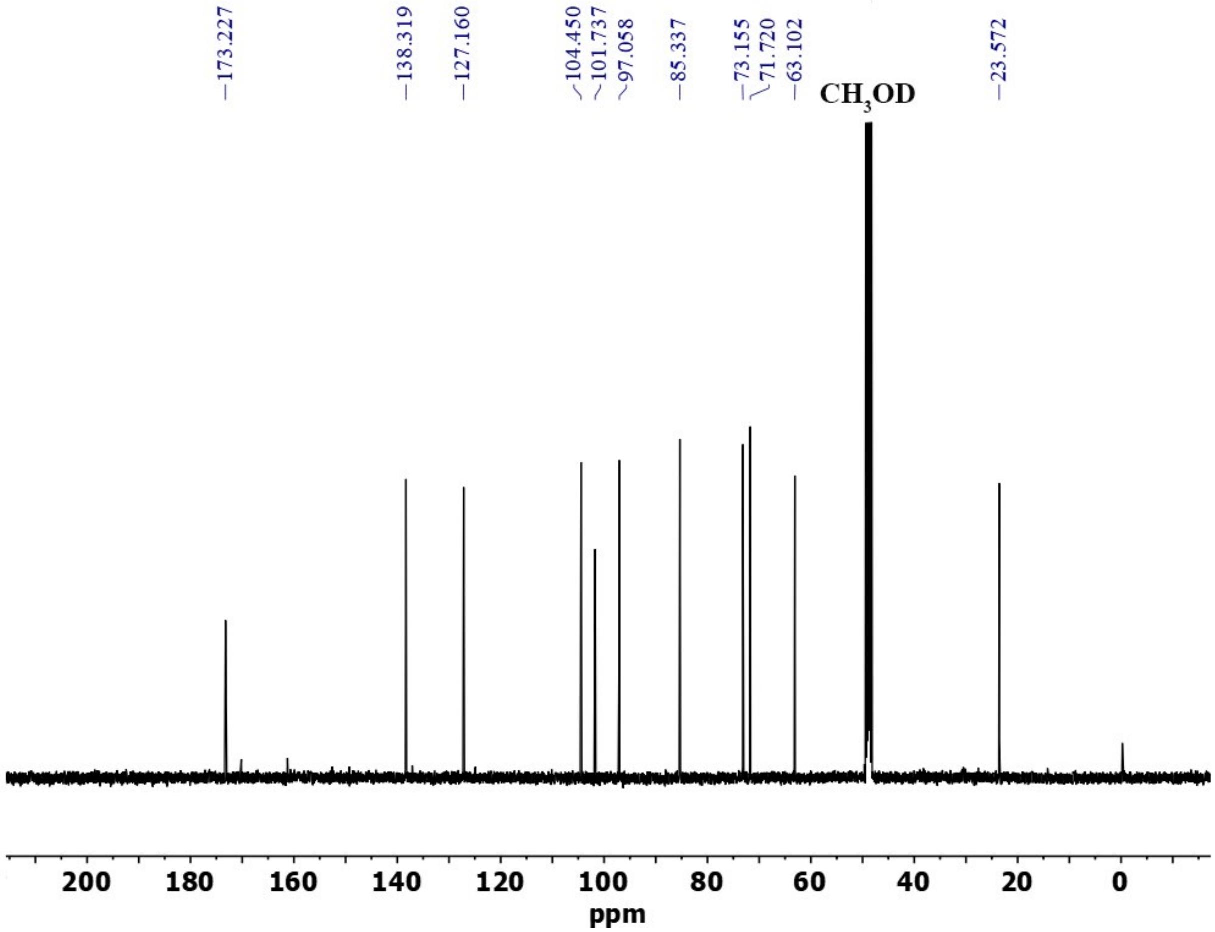

Figure S9. ^{13}C NMR Spectrum of NRH in CD_3OD.

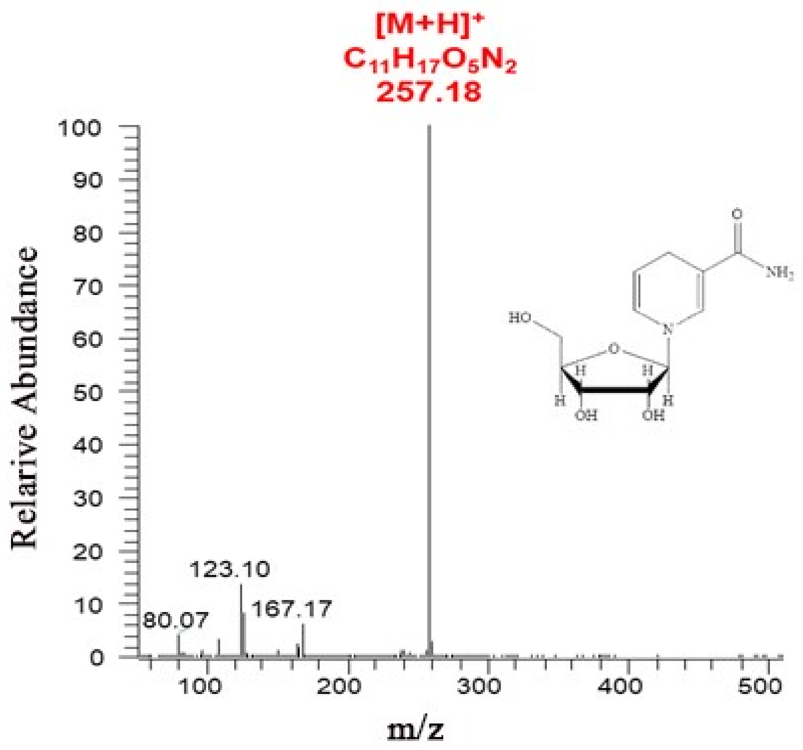

Figure S10. MS of NRH.

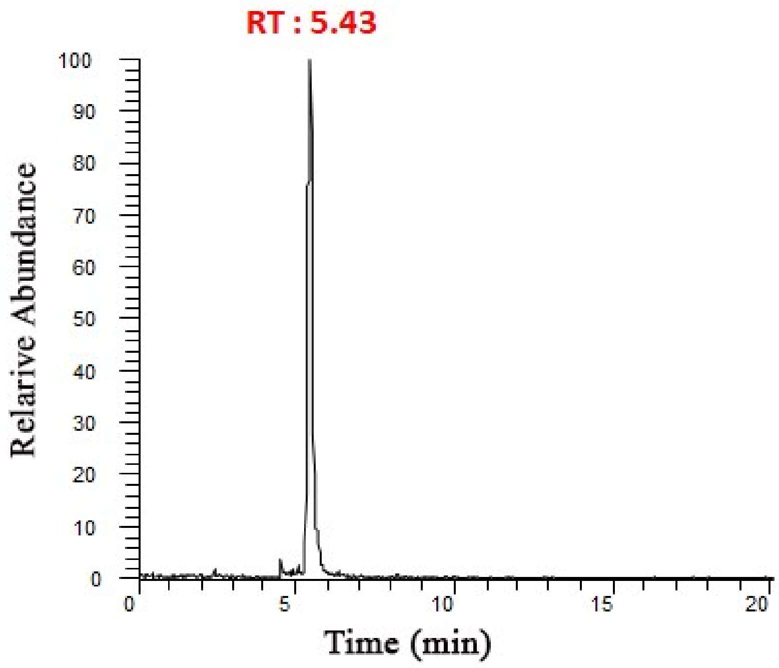

Figure S11. Selected Reaction Monitoring (SRM) of NRH.

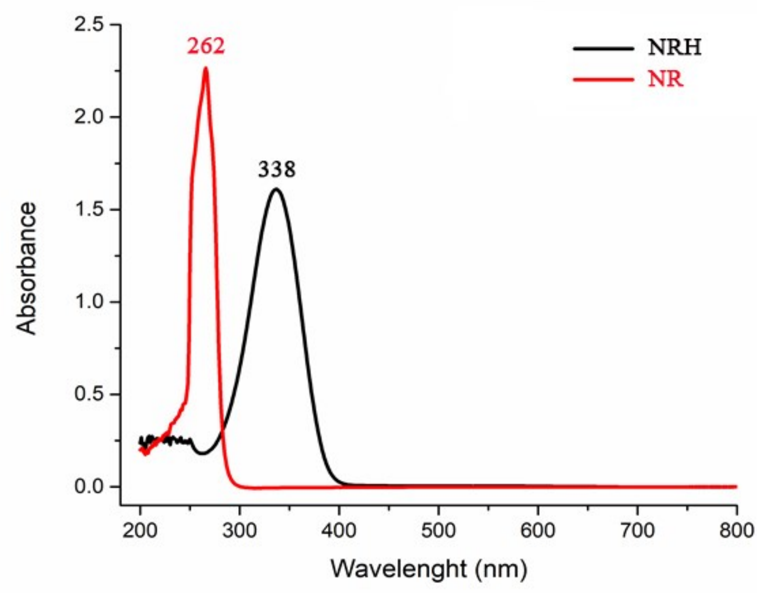

Figure S12. UV-VIS Spectrum of NRH in H_2O.

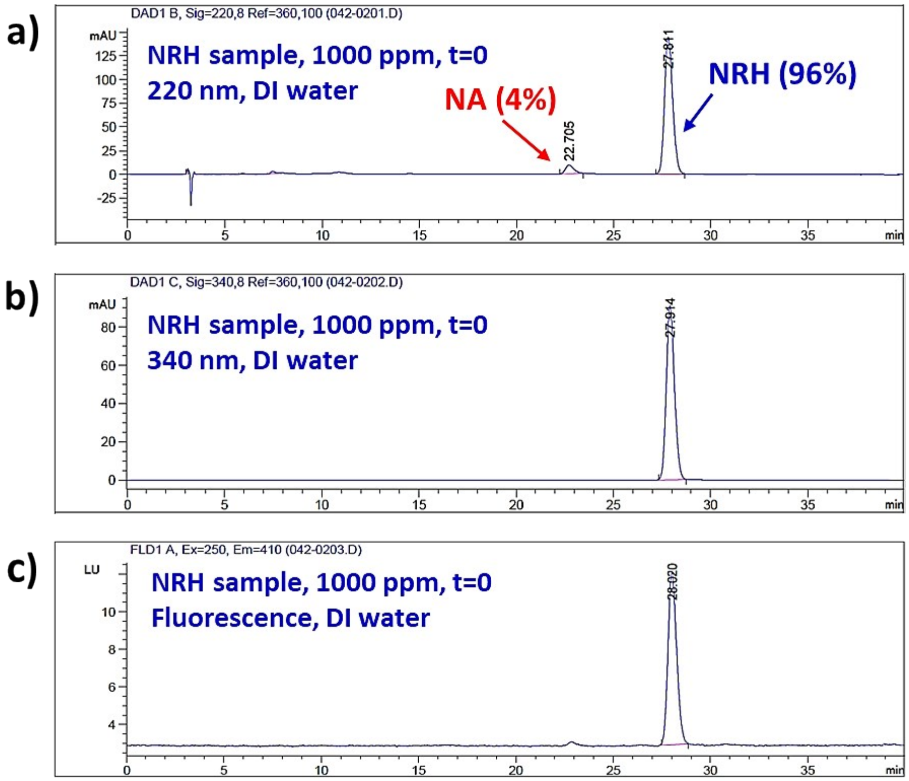

Figure S13. The HPLC chromatograms of the purified NRH **a**) UV detector at 220 nm, **b**) UV detector at 340 nm, and **c**) fluorescence detector.

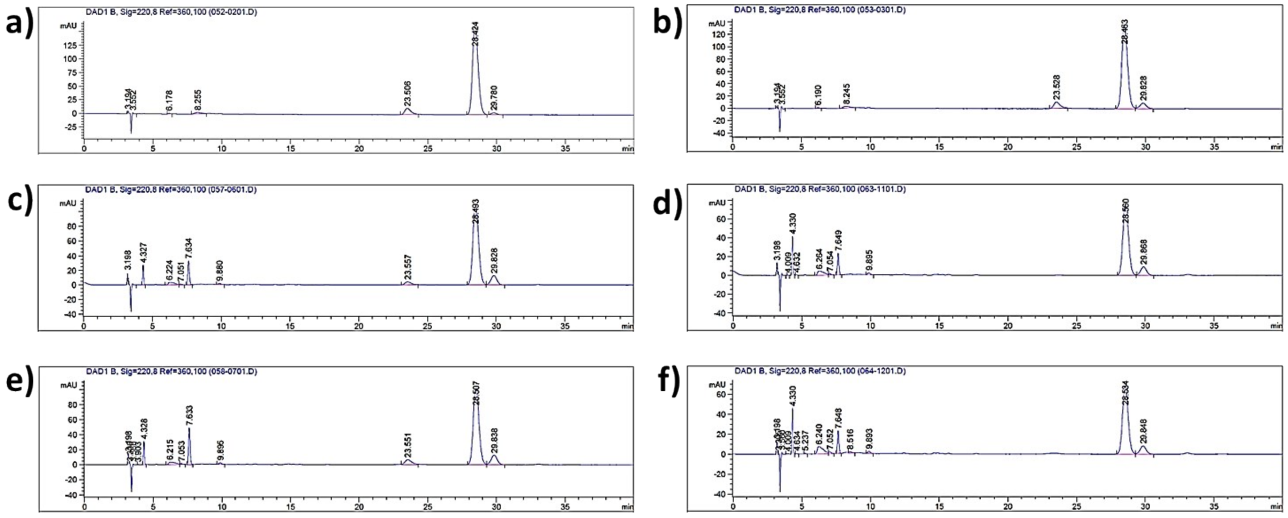

Figure S14. The HPLC chromatograms of the NRH aqueous solutions stored at different conditions for 60 days. **a**) at 4 °C under N_2 and darkness, **b**) at 4 °C under air and darkness, **c**) at 25 °C under N_2 and darkness, **d**) at 25 °C under air and darkness, **e**) at 25 °C under N_2 and light, and **f**) at 25 °C under air and light.

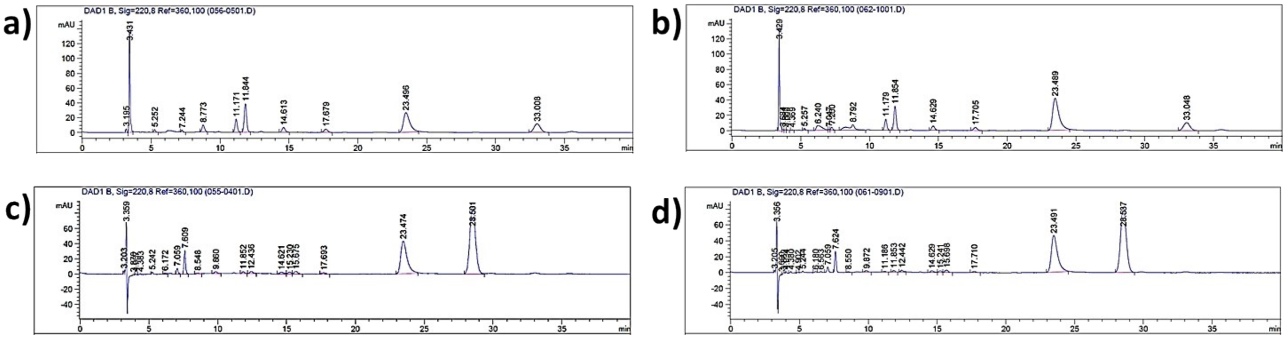

Figure S15. The HPLC chromatograms of the NRH aqueous solutions stored at different conditions for 60 days. **a**) pH= 7.0, at 25 °C, under N2 and dark, **b**) pH= 7.0, at 4 °C, under air and dark, **c**) pH= 9.0, at 25 °C, under N2 and dark, and **d**) pH= 9.0, at 25 °C, under air and dark.

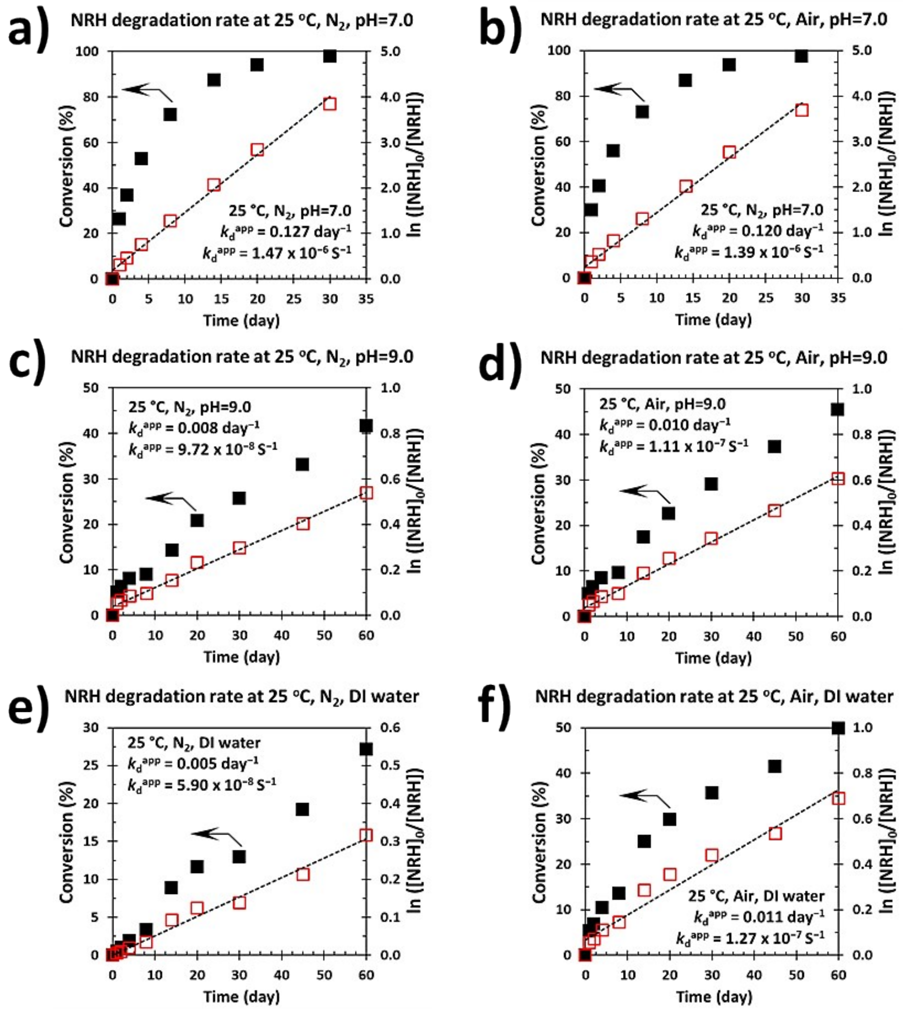

Figure S16. Kinetic graphs and the first order degradation rates for some of NRH samples. **a, b**) pH= 7.0, at 25 °C, under N_2 and air, **c, d**) pH= 9.0, at 25 °C, under N_2 and air, **e, f**) DI water, at 25 °C, under N2 and air.
